# Case of Cataract of Both Eyes,—Absorption of a Portion of the One Following an Operation on the Other, Eighteen Months after the Rupture of the Capsule

**Published:** 1839-01-26

**Authors:** 


*Case of Cataract of both Eyes,—absorption of a portion of the one following an operation on the other, eighteen months after the Rupture of the Capsule.*

Hester H—, aged sixteen years, coloured, was admitted August 2d, 1838, for cataract of both eyes. On her admission, the sight was exceedingly slight, being barely able to distinguish day-light. At this time the right capsule was perfectly opaque, though it had once been operated on. She states that she has had the disease for two years, and that she perceived it coming on by rapid strides, equally, in both eyes, a few months previous to the operation. After attention to her general health, she was operated on by Dr. Norris, on the left eye, three times between the time of her admission and the 15th of October. Soon after the first operation, (that of breaking the capsule,) the cataract of the right eye began to be absorbed, and continued to be so, until a small spot was made, which enabled her to see a little. After the last operation on the left eye, absorption of the capsule of it ceased for some time.

*Nov. 14th.*—Dr. Harris operated on the right, by Saunders' method, introducing the needle through the pupil. Absorption did not take place for some days, though little inflammation followed.

On the 28th she was again operated on, on the right side, when a strong fibrous portion of the capsule was found extending across the pupil, which resisted all efforts to divide it. After this, the left eye was operated on, and the girl put under the internal use of iodine, as well as of a wash of Lugol's solution of iodine and laudanum.

Under this treatment, the eyes improved rapidly, and she is now, January 22d, free from any obstruction of vision in the left eye, and also in the right, with the exception of the band before spoken of.

---

On the 16th of January, Dr. Randolph presented to the class the patient from whom he had removed the *parotid gland*. The wound had then entirely healed, except a point at the lower extremity, where a small ligature was still remaining. The ligature on the carotid came away January 5th; the patient had not had a bad symptom since the operation, and the motion in the parts was returning. Since that date, the last ligature came off, and the wound has now, January 22d, 1839, perfectly healed and he is recovering from the paralysis of the eye-lid.

This patient died suddenly a short time after leaving the Hospital [illegible]

